# Dynamic Prognostic Nutritional Index With Circulating Tumor DNA Predicts Survival in Localized Pancreatic Ductal Adenocarcinoma

**DOI:** 10.1002/jso.70296

**Published:** 2026-06-02

**Authors:** Alex Horowitz, Claire Shen, Dominic J. Vitello, Madison Cox, Amy Wells, Krishay Sridalla, David J. Bentrem, Qiang Zhang, Ishan Roy, Akhil Chawla

**Affiliations:** ^1^ Department of Surgery Division of Surgical Oncology Chicago Illinois USA; ^2^ Northwestern Quality Improvement, Research & Education in Surgery, Department of Surgery Northwestern University Feinberg School of Medicine Chicago Illinois USA; ^3^ Northwestern Medicine Cancer Centers Northwestern Medicine Regional Medical Group Winfield Illinois USA; ^4^ Robert H. Lurie Comprehensive Cancer Center of Northwestern University Chicago Illinois USA; ^5^ Circulating Tumor Cell Core Facility Robert H. Lurie Comprehensive Cancer Center, Northwestern University Feinberg School of Medicine Chicago Illinois USA; ^6^ Department of Physical Medicine & Rehabilitation Northwestern University Feinberg School of Medicine Chicago Illinois USA; ^7^ Shirley Ryan AbilityLab Chicago Illinois USA

**Keywords:** biomarker, circulating tumor DNA, neoadjuvant chemotherapy, pancreatic ductal adenocarcinoma, prognostic nutritional index

## Abstract

**Background and Objectives:**

To evaluate the prognostic significance of baseline and longitudinal changes in prognostic nutritional index (PNI) in localized pancreatic ductal adenocarcinoma (PDAC) and to assess the complementary prognostic value of PNI with tumor burden measured by circulating tumor DNA (ctDNA).

**Methods:**

We analyzed 127 patients with localized PDAC enrolled in a prospective biomarker study (2020–2024). Laboratory values were collected at five standardized timepoints. PNI was calculated as (10 × albumin) + (0.005 × lymphocyte count). *KRAS* ctDNA was assessed using digital droplet PCR. The primary endpoint was overall survival (OS), analyzed using Kaplan–Meier and Cox regression, with longitudinal changes assessed using Friedman testing.

**Results:**

Low PNI (< 45) at diagnosis was associated with worse OS (*p* = 0.043). PNI declined significantly across treatment timepoints (*p* < 0.001). In multivariable analysis, decline in PNI during neoadjuvant chemotherapy independently predicted worse OS (HR 1.095, 95% CI 1.042–1.152, *p* < 0.001). Patients with both low PNI and ctDNA positivity had a 3.64‐fold increased risk of death (HR 3.64, 95% CI 1.32–10.04, *p* = 0.012).

**Conclusions:**

PNI is a dynamic, clinically accessible biomarker that independently predicts survival in localized PDAC. Integration of PNI with ctDNA improves prognostic stratification by capturing both patient host vulnerability and tumor burden.

## 1 Introduction

Pancreatic ductal adenocarcinoma (PDAC) remains a leading cause of cancer mortality despite advances in multimodal care. In localized disease, neoadjuvant chemotherapy (NAC) is increasingly used to improve R0 resection rates and treat micrometastatic disease [1, 2, 3, 4]. Response assessment relies on CA 19‐9 and imaging, but both have limitations, such as secretor status and biologic variability for CA 19‐9, and the difficulty of separating fibrosis from viable tumor on restaging scans [5, 6]. Circulating tumor DNA (ctDNA) provides a complementary, tumor‐derived signal with growing prognostic utility in localized PDAC across platforms, including targeted next‐generation sequencing and droplet digital PCR (ddPCR) [7, 8, 9, 10, 11].

Beyond tumor intrinsic features, there has been an increasing understanding that host‐level factors may influence outcomes. In PDAC and other malignancies, systemic immune, inflammatory, and nutritional status, shapes tolerance of therapy, perioperative risk, and survival [12, 13, 14, 15, 16]. The prognostic nutritional index (PNI), a composite of serum albumin and lymphocyte count, captures nutritional reserve and cellular immunity in a single metric (PNI = 10 × albumin [g/dL] + 0.005 × lymphocytes [/mm^3^]) and has shown prognostic value across gastrointestinal cancers, including PDAC [12, 13, 17, 18, 19]. PNI is appealing as a biomarker because it is inexpensive, reproducible, and biologically anchored in cancer‐related inflammation and cachexia pathways that drive hypoalbuminemia and lymphopenia [12, 13, 14, 15, 16]. Although most studies report static preoperative PNI, early data suggest PNI is dynamic during therapy and may track clinical resilience and tolerance of multimodality treatment [13, 20, 21, 22].

What remains less clear is whether integrating dynamic PNI with ctDNA can improve prognostic stratification in localized PDAC. ctDNA reflects viable tumor burden and biology [7, 8, 9, 10, 11, 23, 24, 25], whereas PNI indexes the immune, inflammatory, and nutritional status of a patient [12, 13, 14, 15, 16, 17, 18, 19]. As PNI and ctDNA markers capture distinct and complementary aspects of disease biology, combined assessment may provide more comprehensive prognostic information than either marker alone.

We therefore evaluated baseline and longitudinal PNI, tested a clinically used cut‐point of PNI < 45 to designate low PNI [15, 17, 18, 19, 20], and examined joint stratification with *KRAS* ctDNA in a prospective single‐institution cohort undergoing standard NAC and surgery. We hypothesized that low PNI associates with worse OS, declining PNI during NAC associates with worse OS, and patients with both low PNI and ctDNA positivity experience the worst outcomes.

## 2 Materials and Methods

### 2.1 Study Design and Population

We performed an analysis of 127 adults with newly diagnosed, localized PDAC enrolled in a prospective biomarker study at Northwestern Medicine between October 2020 and November 2024 (NCT04616131). The study was approved by the Northwestern University Institutional Review Board (NMHC IRB #21‐012) and adhered to Strengthening the Reporting of Observational Studies in Epidemiology (STROBE) guidelines [26]. All cases were reviewed at a multidisciplinary tumor board and managed according to contemporary standards for NAC in localized PDAC [1, 3, 4].

### 2.2 Patient and Treatment Characteristics

Demographic, clinical, laboratory, and treatment data were abstracted from the electronic health record and institutional databases. Resection status and margin were determined by pathology. NAC regimens included mFOLFIRINOX and gemcitabine/nab‐paclitaxel per clinician judgment and patient tolerability, consistent with standard practice [1, 3, 4].

### 2.3 PNI Assessment and Timepoints

PNI was calculated as (10 × serum albumin [g/dL]) + (0.005 × total lymphocytes [/mm^3^]) from CBC and CMP drawn the same day [12, 13, 17, 18, 19, 27]. Five standardized windows (±4 weeks) were targeted: 6 months pre‐diagnosis, diagnosis, after NAC, after surgery, and 6 months after surgery. For group analyses, Low PNI was defined a priori as PNI < 45 and High/Normal PNI as PNI ≥ 45, reflecting common clinical thresholds in the gastrointestinal oncology literature and PDAC‐specific series/meta‐analyses [15, 17, 18, 19, 20, 28]. We chose a PNI of 45 as the low cutoff threshold because it repeatedly discriminates oncologic outcomes, aligns with prior PDAC studies, and balances sensitivity/specificity for adverse events [15, 17, 18, 19, 20, 28]. Using the R package *timeROC*, time‐dependent receiver operating curves (ROC) were used to evaluate PNI < 45 as a model for OS. Area under the curve (AUC) quantified model performance (Table 1).

**Table 1:** Patient demographics.

Characteristic	Overall (*N* = 127)	PNI < 45 (Low)(*N* = 47)	PNI ≥ 45 (normal/high) (*N* = 80)	*p* value
Age (years)				0.5
< 40	1 (0.8%)	0 (0%)	1 (1.3%)	
40–59	19 (15%)	6 (13%)	13 (16%)	
60–69	39 (31%)	13 (28%)	26 (33%)	
70–79	53 (42%)	19 (41%)	34 (43%)	
≥ 80	14 (11%)	8 (17%)	6 (7.5%)	
Sex				0.3
Female	63 (50%)	20 (43%)	43 (54%)	
Male	63 (50%)	26 (57%)	37 (46%)	
BMI at diagnosis (kg/m^2^)	26.3 [23.6, 31.7]	25.6 [22.8, 31.2]	27.4 [24.4, 31.8]	0.2
Clinical stage (AJCC 8th)				0.1
IA	20 (16%)	5 (11%)	15 (19%)	
IB	56 (44%)	26 (55%)	30 (38%)	
II	21 (17%)	9 (19%)	12 (15%)	
III	30 (24%)	7 (15%)	23 (29%)	
Tumor size on imaging (cm)	2.80 [2.20, 3.60]	3.00 [2.30, 3.60]	2.70 [2.00, 3.60]	0.4
Insurance status				0.3
Private	25 (20%)	7 (15%)	18 (23%)	
Underinsured	102 (80%)	40 (85%)	62 (78%)	
Smoking history (ever)				> 0.9
Ever	63 (50%)	23 (50%)	40 (50%)	
Never	63 (50%)	23 (50%)	40 (50%)	
Type 2 diabetes mellitus	50 (39%)	17 (36%)	33 (41%)	0.6
Hypothyroidism	19 (15%)	9 (19%)	10 (13%)	0.3
Chronic kidney disease	5 (3.9%)	2 (4.3%)	3 (3.8%)	> 0.9
Distance to hospital (miles)	9 [5,14]	9 [3,13]	9 [5,15]	0.2
ctDNA detectable (ddPCR)				0.4
Detected	58 (67%)	24 (73%)	34 (64%)	
Not detected	28 (33%)	9 (27%)	19 (36%)	
Radiographic resectability (NCCN)				0.02
Resectable	47 (37%)	18 (38%)	29 (36%)	
Borderline resectable	57 (45%)	26 (55%)	31 (39%)	
Unresectable	23 (18%)	3 (6.4%)	20 (25%)	
Underwent surgical resection				0.96
Yes	72 (57%)	26 (55%)	46 (58%)	
No	55 (43%)	21 (45%)	34 (43%)	
Resection margins				0.69
R0	69 (96%)	25 (96%)	44 (96%)	
R1	1 (1%)	0 (0%)	1 (2%)	
R2	2 (3%)		1 (2%)	
Surgical procedure				0.42
Pancreaticoduodenectomy	52 (72%)	21 (81%)	31 (67%)	
Subtotal pancreatectomy	20 (28%)	5 (19%)	15 (33%)	

*Note:* Percentages represent the proportion of patients within each category, calculated using the cohort size as the denominator. Minor discrepancies from 100% are due to missing data. Discrepancies between the total cohort and the number of individuals with PNI values are due to some patients not undergoing CBC/CMP testing at diagnosis.

Abbreviations: CI, confidence interval; ctDNA, circulating tumor DNA; HR, hazard ratio; NAC, neoadjuvant chemotherapy; NCCN, National Comprehensive Cancer Network; PDAC, pancreatic ductal adenocarcinoma; PNI, prognostic nutritional index.

### 2.4 ctDNA Analysis

We measured *KRAS* mutant ctDNA using the validated platform ddPCR consistent with evolving institutional practice. Plasma was processed under preanalytic standards (double‐spin within institutionally defined time limits, cfDNA extraction from EDTA plasma). *KRAS* hotspot assays (i.e., G12D/V/R) were run on a droplet partitioning platform with appropriate positive/negative controls. A sample was considered positive when the mutant droplet count exceeded the platform threshold and met prespecified assay acceptance criteria; variant allele frequency reporting followed manufacturer and lab standard operating procedures [9, 14, 23, 29]. ddPCR enables absolute quantification with high analytic sensitivity through massive single‐molecule partitioning [14].

### 2.5 Statistical Analysis

Analyses used R via RStudio (version 2024.04.2 + 764). Baseline characteristics were summarized overall and by PNI group (Low < 45 vs. High/Normal ≥ 45). Continuous variables were described using median (IQR) and compared with Wilcoxon rank sum or Kruskal–Wallis as appropriate; categorical variables were described using *n* (%) and compared using *χ* ^2^ tests when cell counts permitted, otherwise using Fisher's exact test. Correlations used Spearman's *ρ*. Longitudinal PNI was assessed using Friedman tests with Bonferroni‐adjusted Wilcoxon signed rank post hoc comparisons. Survival was analyzed by Kaplan–Meier with log‐rank tests and by Cox univariable and multivariable regressions. Multivariable models adjusted for age, AJCC 8th edition stage, tumor size, smoking history, CA 19‐9, and ctDNA status [5, 7, 8, 9, 10, 11]. Two‐sided *p* < 0.05 was considered significant. Reporting follows STROBE guidelines [26].

## 3 Results

### 3.1 Patient and Treatment Characteristics

The cohort included 127 patients (Table I). Most patients were aged 70–79 years (42%). Sex distribution and smoking history were evenly balanced. Median BMI at diagnosis was 26.3 kg/m^2^ (IQR 23.6–31.7). Type 2 diabetes mellitus was present in 39% of patients as the major comorbidity at the time of diagnosis. Clinical stage at diagnosis based on AJCC 8th edition was IA in 15%, IB in 46%, II in 13%, III in 23%, and IV in 2.5% of patients. Insurance grouping showed that 80% of the cohort were categorized as having Medicare, Medicaid, or no insurance, and 20% had private insurance. Median distance from residence to the treating center was 9 miles (IQR 5–14). Patients received guideline‐concordant NAC and were evaluated at standardized laboratory windows [1, 3, 4]. At diagnosis, ddPCR‐based ctDNA testing was available for 86/127 (67%) of patients, with *KRAS* mutant ctDNA detected in 58/86 (67%).

Across the low PNI (< 45) and high/normal PNI (≥ 45) groups, most baseline and clinical characteristics were similar; however, radiographic resectability differed significantly between groups, with the low PNI cohort containing proportionally fewer unresectable tumors and a greater representation of borderline resectable disease, whereas the high/normal PNI group demonstrated a more even distribution across categories (Table 2).

**Table 2:** Multivariable Cox regression for predictors of overall survival.

Characteristic	HR	95% CI	*p*‐value
ΔPNI (Diagnosis to after NAC, per point decline)	1.10	1.04–1.15	< 0.001
Age (years)	1.05	1.01–1.09	0.024
Tumor size on imaging (cm)	1.32	0.83–2.10	0.2
Ever smoker	0.56	0.25–1.24	0.2
Clinical stage			
IA	0.56	0.06–5.51	0.6
IB	0.61	0.07–5.16	0.6
II	2.67	0.28–25.5	0.4
III	0.43	0.04–4.61	0.5
CA19‐9 at diagnosis (per 1 U/mL)	1.00	1.00–1.00	0.003
ctDNA KRAS positive at diagnosis (ddPCR)	2.66	0.97–7.27	0.057

Abbreviations: CI, confidence interval; ctDNA, circulating tumor DNA; HR, Hazard ratio; NAC, Neoadjuvant chemotherapy; NCCN, National Comprehensive Cancer Network; PDAC, Pancreatic ductal adenocarcinoma; PNI, Prognostic nutritional index.

### 3.2 Baseline PNI and Survival

**Figure 1:**
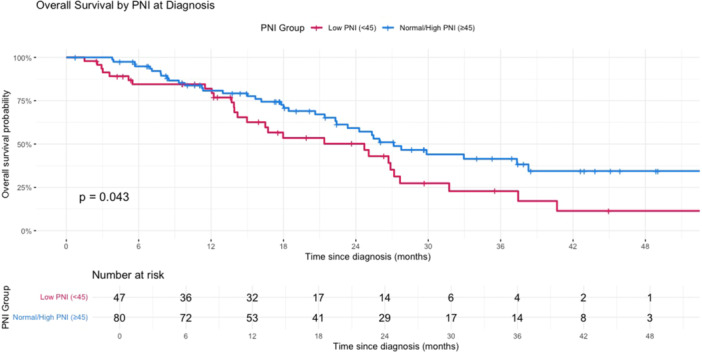
Kaplan–Meier survival curves by baseline PNI (< 45 vs. ≥ 45). Lower PNI is associated with inferior OS (log‐rank *χ* ^2^ = 4.09, *p* = 0.043). ctDNA, circulating tumor DNA; NAC, neoadjuvant chemotherapy; OS, overall Survival; PDAC, pancreatic ductal adenocarcinoma; PNI, prognostic nutritional index.

At diagnosis, the cohort had a median PNI of 48.5. Patients with low PNI (< 45) had a median PNI of 40.0 (mean 37.3; range 11.4–44.5; *n* = 47), whereas those with normal/high PNI (≥ 45) had a median PNI of 50.5 (mean 51.1; range 45–61; *n* = 80). Median follow‐up by reverse Kaplan–Meier was 29.7 months, with a maximum observed follow‐up of 53.4 months. Median overall survival (OS) differed between groups: 24.7 months in the low PNI group versus 27.1 months in those with normal/high PNI with a significant separation in Kaplan–Meier survival curves between PNI groups (log‐rank *χ* ^2^ = 4.09, *p* = 0.043; Figure 1). In a univariable Cox model patients with normal/high PNI (≥ 45) had a 40% lower hazard of death compared with those with low PNI (HR 0.60, 95% CI 0.37–0.99, *p* = 0.045). However, when PNI at diagnosis was modeled as a continuous variable, or when low PNI at diagnosis was modeled as a categorical variable, and adjusted for age, tumor size, smoking history, clinical stage, CA 19‐9, and ctDNA status, baseline PNI was not independently associated with overall survival.

PNI < 45 demonstrated limited discriminatory ability for OS, with time‐dependent AUCs for 0.490, 0.531, and 0.598 at 12, 24, and 36 months, respectively. Modeling PNI as a continuous variable yielded slightly improved discrimination (AUCs 0.511, 0.582, and 0.626), although overall predictive performance remained modest (Supplemental Figure 1).

### 3.3 Pre‐Diagnostic Decline in PNI

Among the 44 patients with PNI values available at both 6 months prior to diagnosis and at diagnosis, PNI declined significantly during the pre‐diagnostic interval. Median PNI decreased from 49.8 (IQR 46.0–54.6) 6 months prior to diagnosis to 48.3 (IQR 42.5–51.6) at diagnosis. The median change in PNI was −2.63 points (IQR −6.63 to +2.00), and the Wilcoxon signed‐rank test confirmed a statistically significant decline (V = 662.5, *p* = 0.0085). However, PNI measured 6 months prior to diagnosis was not associated with overall survival (HR 0.97, 95% CI 0.90–1.05, *p* = 0.48), nor was the magnitude of change in pre‐diagnostic decline in PNI (ΔPNI) significantly associated with survival (HR 0.96, 95% CI 0.90–1.02, *p* = 0.16), suggesting that while early deterioration is detectable, the absolute nutritional immune status at diagnosis carries the primary prognostic signal.

### 3.4 Longitudinal Changes in PNI

We next evaluated the trajectory of PNI across multimodality treatment. Among patients with PNI measured at diagnosis, after NAC, and after surgery (*n* = 67), there were significant differences across timepoints (Friedman *χ* ^2^ = 12.20, df = 2, *p* = 0.0022). Pairwise comparisons demonstrated no significant change from diagnosis to post‐NAC (*p* = 0.85), but a significant decline between post‐NAC and post‐surgery (*p* = 0.00067), indicating that the major nutritional deterioration occurred after surgery rather than during chemotherapy.

**Figure 2:**
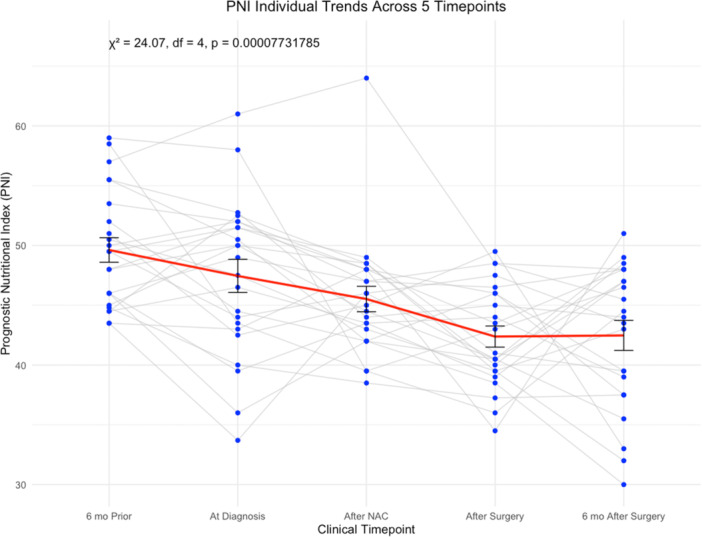
Longitudinal PNI at 6 months pre‐diagnosis, diagnosis, post‐NAC, post‐surgery, and 6 months post‐surgery (Friedman *χ* ^2^ = 24.1, df = 4, *p* < 0.0001). ctDNA, circulating tumor DNA; NAC, neoadjuvant chemotherapy; OS, overall Survival; PDAC, pancreatic ductal adenocarcinoma; PNI, prognostic nutritional index.

In the subset of patients with complete longitudinal data at five clinical timepoints (6 months prior to diagnosis, diagnosis, after NAC, after surgery, and 6 months post‐resection; *n* = 23), PNI also changed significantly across the full treatment course (Friedman *χ* ^2^ = 24.1, df = 4, *p* = 7.7 × 10^−5^; Figure 2). Pairwise analyses showed that PNI significantly declined by the end of NAC relative to the pre‐diagnosis value (*p* = 0.028), fell further after surgery (*p* = 0.027), and, despite partial postoperative recovery, remained significantly lower than both pre‐diagnosis (*p* = 0.0049) and diagnosis (*p* = 0.011) values at 6 months after surgery. These findings indicate that PNI remains relatively stable through NAC but declines substantially after surgical resection, with incomplete recovery by 6 months postoperatively.

### 3.5 Change in PNI during NAC and Survival

We next evaluated whether the magnitude of PNI decline during NAC was prognostic. Among 92 patients with PNI measured at diagnosis and after NAC, with a median follow‐up by reverse Kaplan–Meier of 29.7 months, each 1‐point decline in PNI was associated with a 3.7% increased hazard of death on univariable analysis (HR = 1.037, 95% CI 1.003–1.074, *p* = 0.036). In multivariable modeling adjusting for age, tumor size on imaging, smoking history, clinical stage, CA 19‐9 at diagnosis, and ctDNA *KRAS* positivity by ddPCR, change in PNI remained independently associated with overall survival. Each 1‐point PNI decline conferred a 9.5% increase in the hazard of death (adjusted HR = 1.095, 95% CI 1.042–1.152, *p* < 0.001; Table II). For the clinical context, a 5‐point decline in PNI corresponded to approximately a 1.6‐fold higher hazard of death, and a 10‐point decline corresponded to a 2.5‐fold higher hazard. While median decrease in PNI was modest at 1.5‐points between diagnosis and NAC, 35.9% (33/92) of patients with an available CBC/CMP at diagnosis and after NAC had a 5‐point or greater drop in PNI with 7.6% (7/92) having a drop of greater than 10 points, change in PNI may identify a particularly at‐risk group with depleted nutritional reserve. Taken together, the magnitude of nutritional decline during NAC is a strong and independent predictor of overall survival, outperforming baseline PNI measures and retaining significance even after adjusting for key tumor and host related factors.

### 3.6 Joint Stratification by PNI and CtDNA

**Figure 3:**
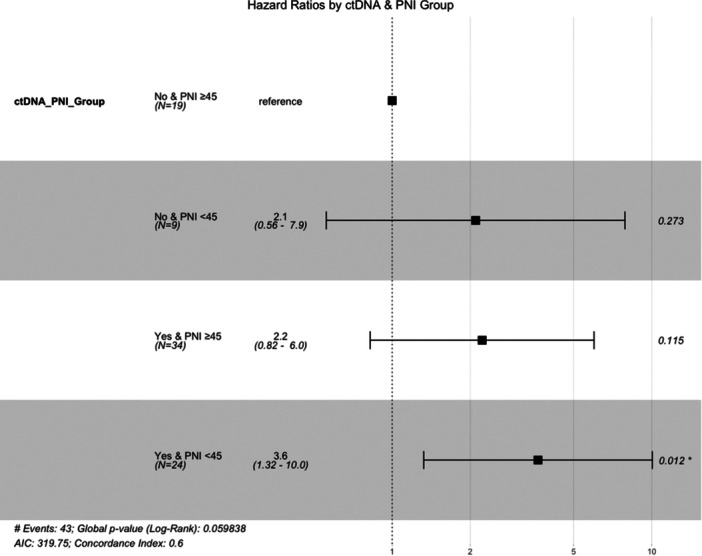
Overall‐survival hazard ratios by ctDNA (detectable vs. not) and PNI (low < 45 vs. high ≥ 45) groups; low PNI with detectable ctDNA shows 3.64 fold higher risk (HR 3.64, 95% CI 1.32–10.04, *p* = 0.012). ctDNA, circulating tumor DNA; NAC, neoadjuvant chemotherapy; OS, overall survival; PDAC, pancreatic ductal adenocarcinoma; PNI, prognostic nutritional index.

We then evaluated whether integrating host nutritional status with tumor‐derived ctDNA improved risk stratification using ddPCR‐based *KRAS* ctDNA. Using a PNI cutoff of 45, patients were classified into four groups based on baseline PNI (≥ 45 vs. < 45) and ctDNA *KRAS* status by ddPCR (positive vs negative). Compared with the reference group of ctDNA‐negative patients with PNI ≥ 45, those with low PNI but negative ctDNA had a numerically higher but not statistically significant hazard of death (HR 2.10, 95% CI 0.56–7.88, *p* = 0.27; Figure 3). Patients with detectable *KRAS* ctDNA but preserved PNI (≥ 45) also demonstrated a nonsignificant trend toward worse survival (HR 2.22, 95% CI 0.82–5.99, *p* = 0.12). The highest risk group consisted of patients with both low PNI and ctDNA positivity, who experienced a significantly increased hazard of death relative to the reference group (HR 3.64, 95% CI 1.32–10.04, *p* = 0.012). The global likelihood ratio test for the four level PNI/ctDNA grouping approached but did not meet statistical significance (*p* = 0.06), suggesting a stepwise pattern in which combined host and tumor vulnerability identifies a clinically meaningful high‐risk subgroup despite limited sample size.

To evaluate whether the prognostic effect of PNI at diagnosis differed by ctDNA status, we tested for statistical interaction between baseline PNI and KRAS positivity by ddPCR. In univariable modeling, the interaction term was not significant (HR 1.027, 95% CI 0.946–1.114, *p* = 0.53), indicating no evidence that the association between PNI and survival varied by ddPCR status. In adjusted analysis controlling for age, tumor size, smoking history, and clinical stage, the interaction term remained similar (HR 1.087, 95% CI 0.995–1.188, *p* = 0.064), suggesting no effect modification. These results indicate that while PNI and ctDNA are individually and jointly prognostic, there is no definitive statistical interaction between them, and their prognostic contributions appear additive.

## 4 Discussion

In this study, we evaluated the prognostic nutritional index (PNI), a composite marker of systemic inflammatory and nutritional status, in patients with localized PDAC treated with NAC and surgery. We demonstrated that PNI is a dynamic biomarker that evolves across the treatment continuum, with significant deterioration beginning prior to diagnosis. While low baseline PNI was associated with worse overall survival, it did not retain independent prognostic significance after adjustment for tumor and clinical factors. In contrast, the magnitude of decline in PNI during neoadjuvant therapy (ΔPNI) emerged as a strong independent predictor of survival, outperforming static measurements. Furthermore, integrating PNI with KRAS mutant circulating tumor DNA (ctDNA) enabled additive risk stratification, identifying a high‐risk subgroup characterized by both host vulnerability and tumor burden. Together, these findings support a model in which dynamic host resilience, rather than baseline status alone, plays a critical role in determining outcomes in localized PDAC.

The association between low baseline PNI and worse OS is consistent with prior work demonstrating that hypoalbuminemia and lymphopenia reflect diminished immunologic competence, systemic inflammation, and impaired nutritional reserve in patients with PDAC [12, 13, 14, 15, 16, 17, 18, 19]. Patients with PNI < 45 experienced shorter median survival and a higher hazard of death on univariable analysis, supporting the clinical relevance of this commonly used threshold. Particularly given that the low PNI group in our cohort contained fewer unresectable patients, the poorer survival of that group cannot be explained by anatomical tumor differences, which further emphasizes the poor prognosis associated with PNI < 45. This may additionally indicate that PNI is a robust prognostic measure in resectable and borderline resectable disease populations. However, baseline PNI did not remain independently prognostic after multivariable adjustment, suggesting that a single timepoint measurement may be insufficient to capture the dynamic physiologic changes occurring during treatment. This finding aligns with emerging data indicating that static inflammatory and nutritional markers may be confounded by tumor burden, comorbidities, and treatment selection, thereby limiting their independent predictive value [13, 14, 15, 16]. The limited discriminatory ability as measured by AUCs for PNI < 45 and continuous baseline PNI additionally reflects this observation, suggesting that it may be insufficient as a standalone indicator without dynamic measurements or integration with tumor‐derived markers such as ctDNA. In contrast, longitudinal changes in these markers may better reflect a patient's ability to tolerate therapy and adapt to systemic stress. Future studies incorporating inflammatory and cachexia‐associated biomarkers, including circulating cytokines (e.g., IL‐6, TNF‐α, and IL‐1β) and body composition or sarcopenia metrics, may further refine individualized prognostic modeling by capturing complementary aspects of tumor burden, systemic inflammation, and host metabolic reserve. Taken together, our results suggest that while baseline PNI provides an initial snapshot of host status, it is the trajectory of PNI over time that more accurately captures clinically meaningful vulnerability in localized PDAC.

Beyond baseline associations, our findings demonstrate that PNI declines during the pre‐diagnostic period, prior to clinical recognition of disease. Median PNI declined by 2.6 points from 6 months prior to diagnosis to initial presentation (*p* = 0.0085). To our knowledge, no prior PDAC study has characterized pre‐diagnostic PNI trajectories. This pattern is consistent with preclinical cachexia and systemic inflammation, as PDAC induces early metabolic dysregulation and cytokine‐mediated immune suppression driven by IL‐6, TNF‐α, and IL‐1 family cytokines [14, 15, 16]. These paraneoplastic processes reduce albumin synthesis, alter lymphocyte homeostasis, and promote catabolism [14, 15, 16]. While pre‐diagnostic decline was not independently prognostic, it may represent an early marker of tumor–host interaction, warranting further investigation.

Our longitudinal analyses demonstrate that PNI evolves across the treatment course. PNI remained stable from diagnosis through NAC but declined significantly after surgery, with incomplete recovery by 6 months postoperatively. These patterns reflect the cumulative physiologic burden of PDAC‐associated cachexia, exocrine insufficiency, and chronic inflammation. Cytokine‐driven hepatic reprioritization suppresses albumin synthesis while chemotherapy contributes to lymphocyte depletion [14, 15, 16]. Contemporary NAC regimens such as FOLFIRINOX and gemcitabine/nab‐paclitaxel exacerbate anorexia and catabolic stress, and major pancreatic surgery imposes additional metabolic demands. By demonstrating the timing of PNI deterioration, we highlight a perioperative window in which patients may decompensate nutritionally despite appearing stable during systemic therapy [13, 17, 19, 20, 22, 28].

The magnitude of PNI decline during NAC (ΔPNI) emerged as the strongest predictor of OS. Each 1‐point decline conferred a 9.5% increased hazard of death on multivariable analysis, with a 5‐point decline corresponding to a 1.6‐fold higher hazard. Patients undergoing NAC frequently experience multi‐point declines in PNI rather than isolated 1‐point changes, as shown in our cohort, in which over a third of patients experienced a 5‐point or more decline. With incremental decreases leading to compounded risk, this suggests that progressive nutritional deterioration during therapy may reveal a biologically vulnerable subgroup with depleted reserves. This extends prior reports that changes in PNI are more predictive than baseline values [17, 18, 19, 20, 21, 22, 28]. ΔPNI likely integrates worsening systemic inflammation, impaired protein synthesis, lymphocyte exhaustion, and inability to compensate for treatment toxicity [13, 14, 15, 16, 30, 31]. That ΔPNI remained independently prognostic after accounting for CA 19‐9 and ctDNA suggests it captures host resilience not reflected by tumor burden alone. Unlike static measurements, ΔPNI reflects physiologic response to therapy, making it a powerful tool for risk stratification and identifying patients who may benefit from nutritional intervention or treatment modification.

ctDNA has emerged as a biomarker of tumor genomic activity and minimal residual disease in localized PDAC [6, 7, 8, 9, 10, 11, 23, 24, 25, 32]. In contrast, PNI captures host physiology, including systemic inflammation, nutritional reserve, and immune competence [12, 13, 17, 18, 19, 20, 22, 27, 28]. Baseline PNI did not differ by *KRAS* mutant ctDNA status, supporting the independence of these signals. When combined, these markers yielded a four‐level stratification. Patients with preserved PNI (≥ 45) and negative ctDNA had the most favorable outcomes, while those with both low PNI and ctDNA positivity experienced a 3.64‐fold increase in the hazard of death. Although the global test was borderline (*p* = 0.06), the stepwise pattern suggests that host nutritional immune compromise and tumor burden act additively to shape prognosis. Integrated biomarker frameworks that jointly consider tumor biology and host resilience may provide more accurate risk stratification in localized PDAC [6, 7, 8, 9, 10, 11, 13, 23, 24, 25, 32].

Our findings have several clinical implications. Routine calculation of PNI at diagnosis and after NAC could identify patients at elevated risk despite resectable disease [13, 14, 15, 16, 17, 20, 22, 28]. Serial PNI measurement may serve as an early warning for clinically silent nutritional immune decline, prompting targeted support or treatment modifications. ΔPNI could be incorporated into prognostic models alongside ctDNA and CA 19‐9 to refine patient selection for intensified surveillance or clinical trials [1, 2, 3, 4, 6, 7, 8, 9, 10, 11, 13, 23, 24, 25, 32]. Because PNI relies on universally available laboratory parameters, it offers a practical biomarker implementable across diverse practice settings [6, 7, 8, 9, 10, 11, 23, 24, 25, 29, 32].

## 5 Limitations

This single‐institution study reflects evolving treatment paradigms over time, which may limit generalizability. Sample sizes were modest at certain longitudinal timepoints. Although we adjusted for key covariates, residual confounding by unmeasured factors such as sarcopenia and detailed nutritional interventions cannot be excluded. Heterogeneity in chemotherapy regimens and postoperative recovery may influence PNI trajectories. ctDNA assessments using ddPCR may be affected by preanalytic variation and assay‐specific thresholds [7, 8, 9, 10, 11, 21, 24, 25, 31, 32]. As an observational study, our work identifies associations but cannot prove causality. Prospective, multicenter validation and interventional studies are needed to confirm whether modifying PNI trajectories can improve outcomes in localized PDAC.

## 6 Conclusions

In patients with localized PDAC treated with NAC and surgery, the prognostic nutritional index undergoes significant deterioration across the treatment continuum, beginning in the pre‐diagnostic period and accelerating after surgical resection. The magnitude of PNI decline during NAC emerged as a strong independent predictor of overall survival, outperforming static baseline measurements and retaining prognostic significance after adjustment for tumor burden markers, including CA 19‐9 and ctDNA. When integrated with *KRAS* mutant ctDNA status, PNI enabled additive risk stratification, with patients exhibiting both low PNI and detectable ctDNA experiencing markedly worse outcomes. These findings establish ΔPNI as a dynamic, clinically actionable biomarker of host vulnerability that captures physiologic resilience independent of tumor genomic activity. Routine serial assessment of PNI during neoadjuvant therapy may identify patients at elevated risk for poor outcomes despite resectable disease, creating opportunities for targeted nutritional intervention, treatment modification, and refined prognostic counseling. Future prospective studies should evaluate whether interventions aimed at preserving or restoring nutritional immune status can mitigate treatment‐related decline and improve survival in this high‐risk population.

## SYNOPSIS

In this prospective cohort of patients with localized PDAC undergoing NAC and surgical resection, longitudinal declines in prognostic nutritional index independently predicted worse overall survival and were accentuated in patients with detectable ctDNA. Combined assessment of dynamic nutritional status and ctDNA detection provides complementary prognostic information reflecting nutritional reserve and tumor biology.

## Supporting information


**Figure S1:** Time‐dependent ROC for overall survival using (A) PNI < 45 and (B) continuous PNI.

## Data Availability

The data that support the findings of this study are available on request from the corresponding author. The data are not publicly available due to privacy or ethical restrictions.

## References

[1] C. Springfeld , C. R. Ferrone , M. H. G. Katz , et al., “Neoadjuvant Therapy for Pancreatic Cancer,” Nature Reviews Clinical Oncology 20, no. 5 (2023): 318–337, 10.1038/s41571-023-00746-1. 36932224

[2] V. P. Groot , N. Rezaee , W. Wu , et al., “Patterns, Timing, and Predictors of Recurrence Following Pancreatectomy for Pancreatic Ductal Adenocarcinoma,” Annals of Surgery 267, no. 5 (2018): 936–945, 10.1097/SLA.0000000000002234. 28338509

[3] Q. P. Janssen , E. M. O'Reilly , C. H. J. van Eijck , and B. Groot Koerkamp , “Neoadjuvant Treatment in Patients With Resectable and Borderline Resectable Pancreatic Cancer,” Frontiers in Oncology 10 (2020): 41, 10.3389/fonc.2020.00041. 32083002 PMC7005204

[4] A. G. Raufi , G. A. Manji , J. A. Chabot , and S. E. Bates , “Neoadjuvant Treatment for Pancreatic Cancer,” Seminars in Oncology 46, no. 1 (2019): 19–27, 10.1053/j.seminoncol.2018.12.002. 30630600

[5] G. Luo , K. Jin , S. Deng , et al., “Roles of CA19‐9 in Pancreatic Cancer,” Biochimica et Biophysica Acta, Reviews on Cancer 1875, no. 2 (2021): 188409, 10.1016/j.bbcan.2020.188409. 32827580

[6] H. M. H. Diab , H. G. Smith , K. K. Jensen , and L. N. Jørgensen , “The Current Role of Blood‐Based Biomarkers in Surgical Decision‐Making in Patients With Localised Pancreatic Cancer: A Systematic Review,” European Journal of Cancer 154 (2021): 73–81, 10.1016/j.ejca.2021.05.033. 34243080

[7] D. Shah , A. Wells , M. Cox , et al., “Prospective Evaluation of ctDNA by NGS During NAC in Localized PDAC,” Annals of Surgery 281 (2024): 997–1005, 10.1097/SLA.0000000000006209. 38258582 PMC11263501

[8] D. J. Vitello , D. Shah , A. Wells , et al., “Mutant KRAS in ctDNA as a Biomarker in Localized PDAC Treated With NAC,” Annals of Surgery (2024), 10.1097/SLA.0000000000006562. 39471087

[9] M. Huerta , S. Roselló , L. Sabater , et al., “ctDNA Detection by ddPCR in PDAC: Systematic Review,” Cancers 13, no. 5 (2021): 1154, 10.3390/cancers13050994. 33673558 PMC7956845

[10] I. Labiano , A. E. Huerta , M. Alsina , et al., “Building on the Clinical Applicability of ctDNA Analysis in Non‐Metastatic Pancreatic Ductal Adenocarcinoma,” Scientific Reports 14 (2024): 16203, 10.1038/s41598-024-67235-y. 39003322 PMC11246447

[11] J. S. Lee , Y. Han , W. G. Yun , et al., “Parallel Analysis of Pre‐ and Postoperative Circulating Tumor DNA and Matched Tumor Tissues in Resectable Pancreatic Ductal Adenocarcinoma: A Prospective Cohort Study,” Clinical Chemistry 68, no. 12 (2022): 1509–1518, 10.1093/clinchem/hvac153. 36177751

[12] D. Wang , H. Luo , M. Qiu , et al., “Comparison of the Prognostic Values of Various Inflammation Based Factors in Patients With Pancreatic Cancer,” Medical Oncology 29, no. 5 (2012): 3092–3100, 10.1007/s12032-012-0226-8. 22476808

[13] T. Aoyama , Y. Maezawa , I. Hashimoto , Y. Rino , and T. Oshima , “Clinical Impact of Nutrition and Inflammation Assessment Tools in Pancreatic Cancer Treatment,” Anticancer Research 43, no. 9 (2023): 3849–3860, 10.21873/anticanres.16572. 37648333

[14] A. Mantovani , P. Allavena , A. Sica , and F. Balkwill , “Cancer‐Related Inflammation,” Nature 454, no. 7203 (2008): 436–444, 10.1038/nature07205. 18650914

[15] D. C. McMillan , “The Systemic Inflammation‐Based Glasgow Prognostic Score: A Decade of Experience in Patients With Cancer,” Cancer Treatment Reviews 39, no. 5 (2013): 534–540, 10.1016/j.ctrv.2012.08.003. 22995477

[16] K. Shadhu and C. Xi , “Inflammation and Pancreatic Cancer: An Updated Review,” Saudi Journal of Gastroenterology 25, no. 1 (2019): 3–13, 10.4103/sjg.SJG_390_18. 30588953 PMC6373214

[17] S. Itoh , E. Tsujita , K. Fukuzawa , et al., “Prognostic Significance of Preoperative PNI and CA19‐9 for Pancreatic Ductal Adenocarcinoma: A Multi‐Institutional Retrospective Study,” Pancreatology 21, no. 7 (2021): 1356–1363, 10.1016/j.pan.2021.08.003. 34426076

[18] P. Zhao , Z. Wu , Z. Wang , et al., “PNI and Survival After Curative Resection Without NAT: Systematic Review/Meta‐Analysis,” Frontiers in Surgery 9 (2022): 992641, 10.3389/fsurg.2022.992641. 36157419 PMC9500291

[19] M. Ikeguchi , K. Goto , J. Watanabe , et al., “Clinical Importance of Preoperative and Postoperative Prognostic Nutritional Index in Patients With Pancreatic Ductal Adenocarcinoma,” Annals of Hepato‐Biliary‐Pancreatic Surgery 23, no. 4 (2019): 372–376, 10.14701/ahbps.2019.23.4.372. 31825004 PMC6893056

[20] S. Kawahara , T. Aoyama , M. Murakawa , et al., “Prognostic Nutritional Index Is an Independent Risk Factor for Continuing S‐1 Adjuvant Chemotherapy in Patients With Pancreatic Cancer Who Received Neoadjuvant Chemotherapy and Surgical Resection,” BMC Cancer 24, no. 1 (2024): 1469, 10.1186/s12885-024-13244-z. 39609741 PMC11606020

[21] H. Suto , H. Matsukawa , Y. Ando , et al., “Predictive Role of PNI After Neoadjuvant Chemoradiotherapy and Curative Resection,” Journal of Hepato‐Biliary‐Pancreatic Sciences 31 (2024): 404–414, 10.1002/jhbp.1424. 38462668

[22] D. Hackner , S. Merkel , A. Weiß , et al., “Neutrophil‐to‐Lymphocyte Ratio and Prognostic Nutritional Index Are Predictors for Overall Survival After Primary Pancreatic Resection of Pancreatic Ductal Adenocarcinoma: A Single Centre Evaluation,” Cancers 16, no. 16 (2024): 2911, 10.3390/cancers16162911. 39199682 PMC11353046

[23] J. D. Merker , G. R. Oxnard , C. Compton , et al., “Circulating Tumor DNA Analysis in Patients With Cancer: American Society of Clinical Oncology and College of American Pathologists Joint Review,” Journal of Clinical Oncology 36, no. 16 (2018): 1631–1641, 10.1200/JCO.2017.76.8671. 29504847

[24] M. R. Lee , S. M. Woo , M. K. Kim , et al., “Application of Plasma Circulating KRAS Mutations as a Predictive Biomarker for Targeted Treatment of Pancreatic Cancer,” Cancer Science 115, no. 4 (2024): 1283–1295, 10.1111/cas.16104. 38348576 PMC11007020

[25] A. Alqahtani , A. Alloghbi , P. Coffin , C. Yin , R. Mukherji , and B. A. Weinberg , “Prognostic Utility of Preoperative and Postoperative KRAS‐Mutated Circulating Tumor DNA (ctDNA) in Resected Pancreatic Ductal Adenocarcinoma: A Systematic Review and Meta‐Analysis,” Surgical Oncology 51 (2023): 102007, 10.1016/j.suronc.2023.102007. 37852124

[26] E. von Elm , D. G. Altman , M. Egger , S. J. Pocock , P. C. Gøtzsche , and J. P. Vandenbroucke , “The Strengthening the Reporting of Observational Studies in Epidemiology (STROBE) Statement: Guidelines for Reporting Observational Studies,” Lancet 370, no. 9596 (2007): 1453–1457, 10.1016/S0140-6736(07)61602-X. 18064739

[27] A. Tanemura , S. Mizuno , A. Hayasaki , et al., “Onodera's Prognostic Nutritional Index Is a Strong Prognostic Indicator for Patients With Hepatocellular Carcinoma After Initial Hepatectomy, Especially Patients With Preserved Liver Function,” BMC Surgery 20 (2020): 261, 10.1186/s12893-020-00917-2. 33129309 PMC7603728

[28] P. Jiang , X. Li , S. Wang , and Y. Liu , “Prognostic Significance of PNI in Patients With Pancreatic Head Cancer Undergoing Laparoscopic Pancreaticoduodenectomy,” Frontiers in Surgery 9 (2022): 897033, 10.3389/fsurg.2022.897033. 35722527 PMC9198448

[29] B. J. Hindson , K. D. Ness , D. A. Masquelier , et al., “High‐Throughput Droplet Digital PCR System for Absolute Quantitation of DNA Copy Number,” Analytical Chemistry 83, no. 22 (2011): 8604–8610, 10.1021/ac202028g. 22035192 PMC3216358

[30] O. M. Vagnildhaug , D. Blum , A. Wilcock , et al., “The Applicability of a Weight Loss Grading System in Cancer Cachexia: A Longitudinal Analysis,” Journal of Cachexia, Sarcopenia and Muscle 8, no. 5 (2017): 789–797, 10.1002/jcsm.12220. 28627024 PMC5659057

[31] K. Fearon , F. Strasser , S. D. Anker , et al., “Definition and Classification of Cancer Cachexia: An International Consensus,” Lancet Oncology 12, no. 5 (2011): 489–495, 10.1016/S1470-2045(10)70218-7. 21296615

[32] M. Cox , D. J. Vitello , and A. Chawla , “The Current Role of Circulating Tumor DNA in the Management of Pancreatic Cancer,” Journal of Gastrointestinal Cancer 56, no. 1 (2025): 44, 10.1007/s12029-024-01129-0. 39808248

