# Supplementary figures and images for: Dynamic Prognostic Nutritional Index With Circulating Tumor DNA Predicts Survival in Localized Pancreatic Ductal Adenocarcinoma
Source: J Surg Oncol. 2026 Jun 2;134(2):272–81. doi: 10.1002/jso.70296 (PMC13427133; doi:10.1002/jso.70296)

Figure 1A


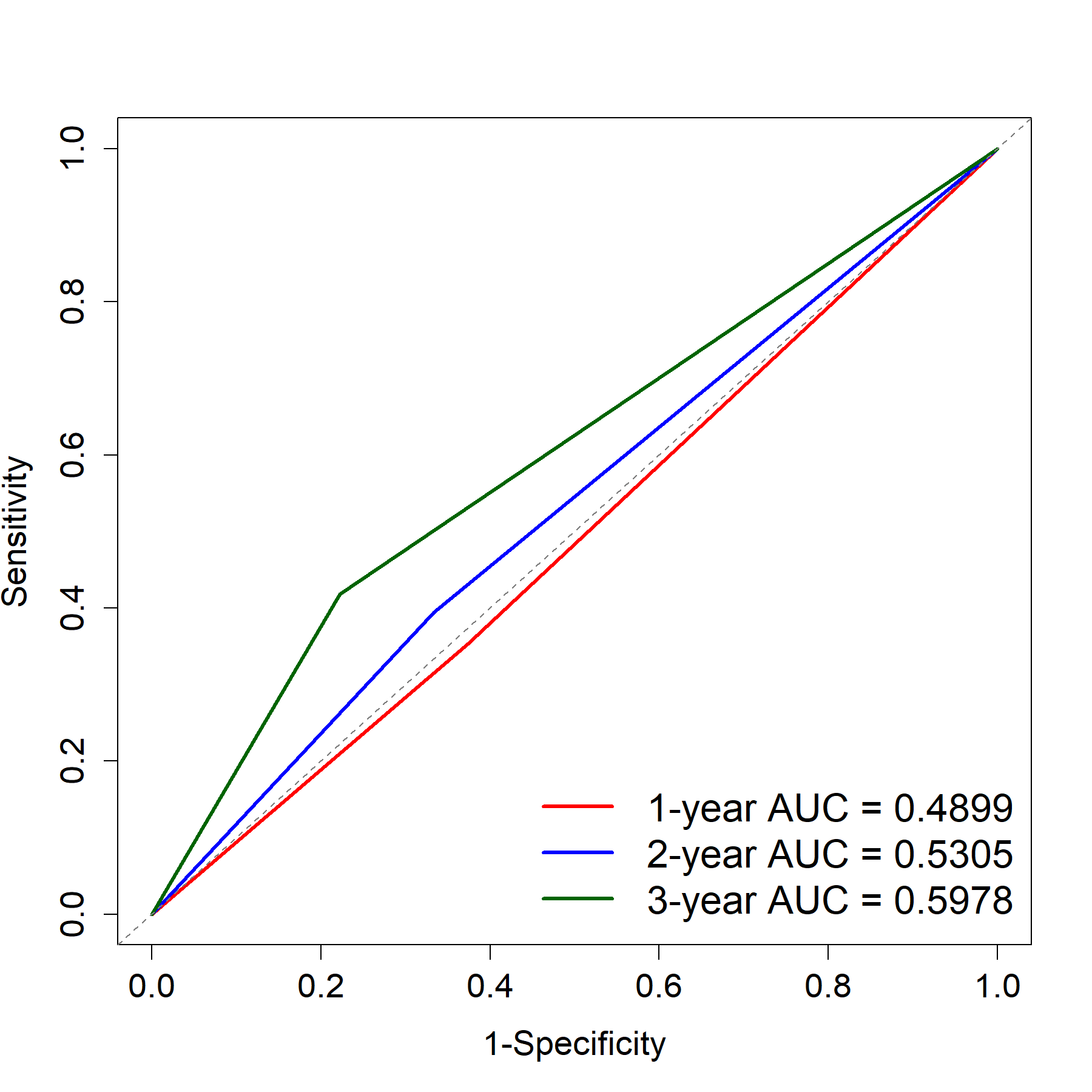


Figure 1B


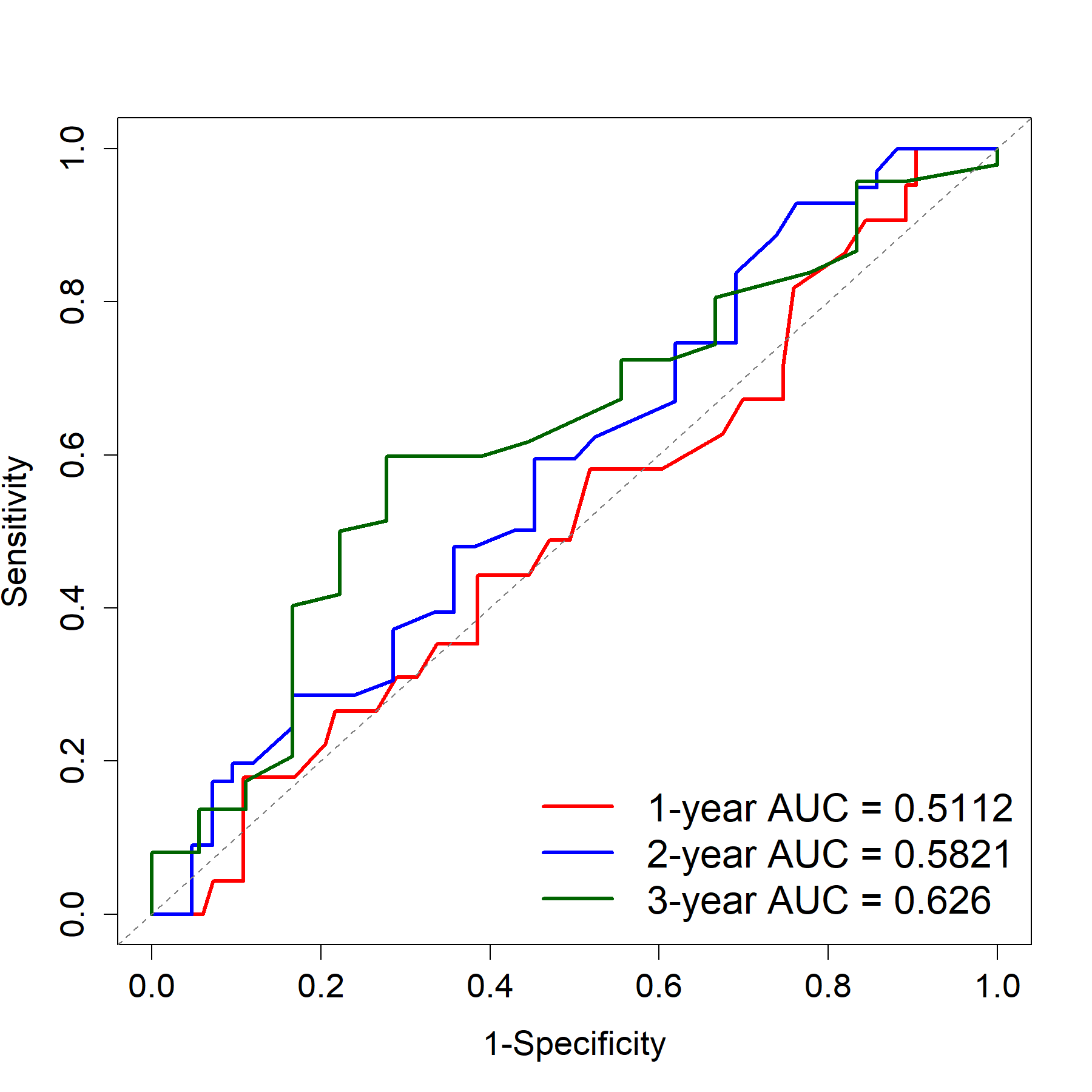

Supplement: Supplementary file 1 — Figure S1: Time‐dependent ROC for overall survival using (A) PNI < 45 and (B) continuous PNI. [file JSO-134-272-s001.docx]
